# Supplementary material for: Stress-induced RNA–chromatin interactions promote endothelial dysfunction
Source: Nat Commun. 2020 Oct 15;11:5211. doi: 10.1038/s41467-020-18957-w (PMC7566596; doi:10.1038/s41467-020-18957-w)
Supplement: Supplementary file 1 — Supplementary Information [file 41467_2020_18957_MOESM1_ESM.pdf]

Supplementary information

Stress-induced RNA-chromatin interactions promote endothelial dysfunction

Calandrelli, R. *et al*.

Supplementary Figures and Tables

Supplementary Figure 1. Venn diagram of up- and down-regulated genes from scRNA-seq. Two-sided Wilcoxon test was used, $P < 9.72e-27$ for Day 3 and $P < 9.93e-115$ for Day 7.

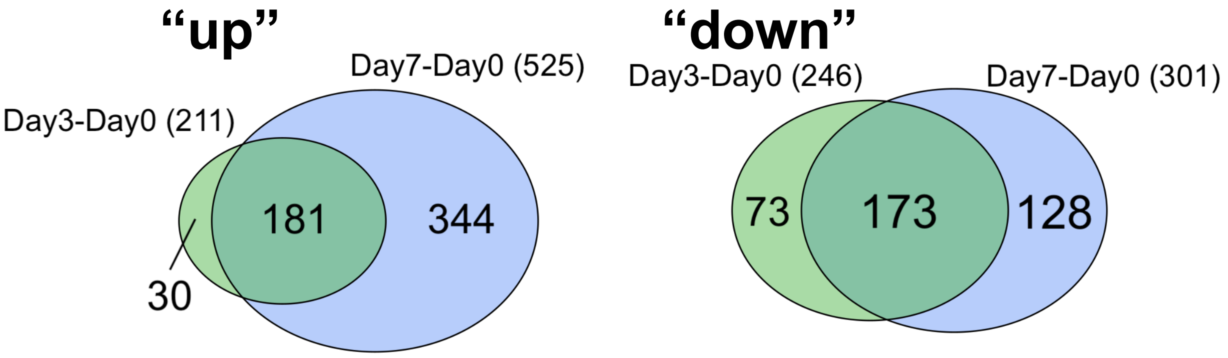

Supplementary Figure 2. HUVECs (at passage 5-6) were kept untreated (NT) or treated with TGF-β (10 ng/ml) and IL-1β (5 ng/ml) (T+I) for 3 or 7 days. Cell morphology was captured by bright-field microscopy. Scale bar = 100 μm. The cell treatment and microscopy were performed 3 times independently with similar observation. Representative images are presented.

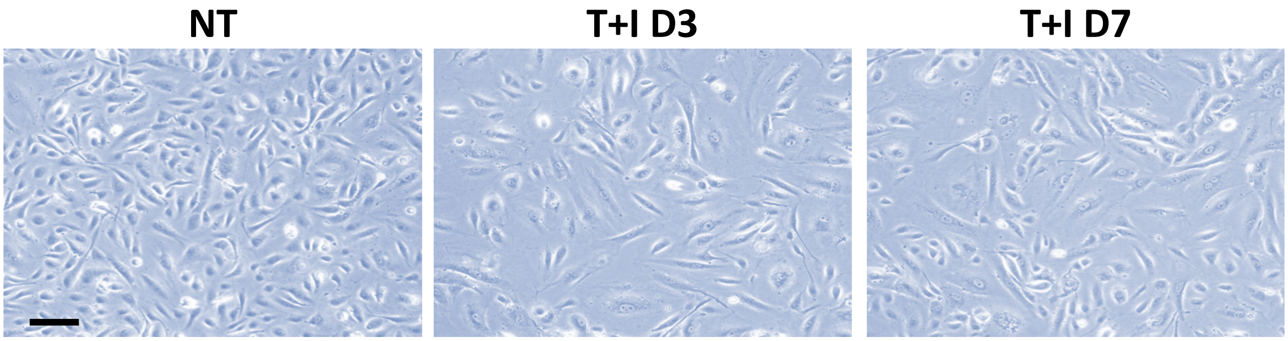

Supplementary Figure 3. Comparison of Hi-C derived genomic features in Day 0 (left), 3 (center), and 7 (right). (a) Pearson correlation matrix of the observed/expected contact matrix, reflecting A/B compartments in red and blue. Bottom: The first eigenvector of the Pearson correlation matrix. Resolution = 1 Mb. (b) Contact matrices of the entire Chromosome 6 at 1 Mb resolution (top row) and of the 80-120 Mb region on Chromosome 6 at 200 kb resolution (bottom row). (c) Measure of Concordance (MoC) between every two time points. The threshold for calling two Hi-C datasets "highly concordant" is MoC > 0.75.

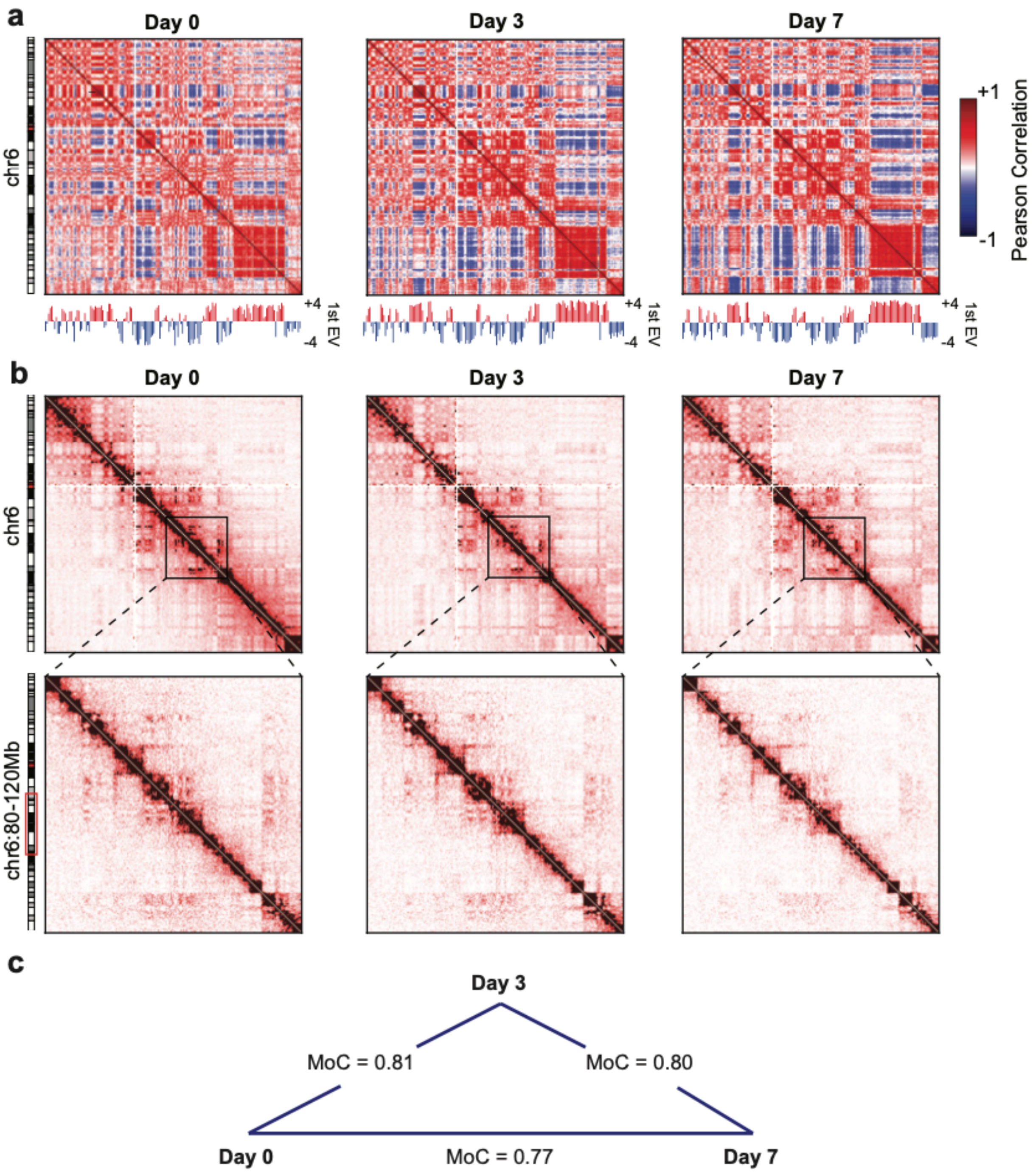

Supplementary Figure 4. iMARGI read pairs and iMARGI derived interactions. (a) Proportions of iMARGI read pairs with the RNA ends (pink) or the DNA ends (green) in Control (Ctrl) and H+T treated (Day 3 and 7 combined) ECs mapped to HUVEC enhancers. Dotted line: the relative size of HUVEC enhancers compared to the size of the genome. (b) Intra- (pink) and inter-chromosomal (green) number of SE pairs at Day 0 and Day 7. (c) Degree distribution (log-log plot) of the SEs in the identified SE RNA-chromatin interactions. The proportions (y axis) of SEs (nodes) in the Day 0 (pink), 3 (green), and 7 (blue) networks are plotted against the degrees of these SEs (x axis). (d,e) Inter- (d) and intra-chromosomal (e) RNA-chromatin interaction networks. Each node is a SE. Each edge is an interaction between two SEs. (f) Number of nodes in inter-chromosomal SE networks. Source data are provided as a Source Data file.

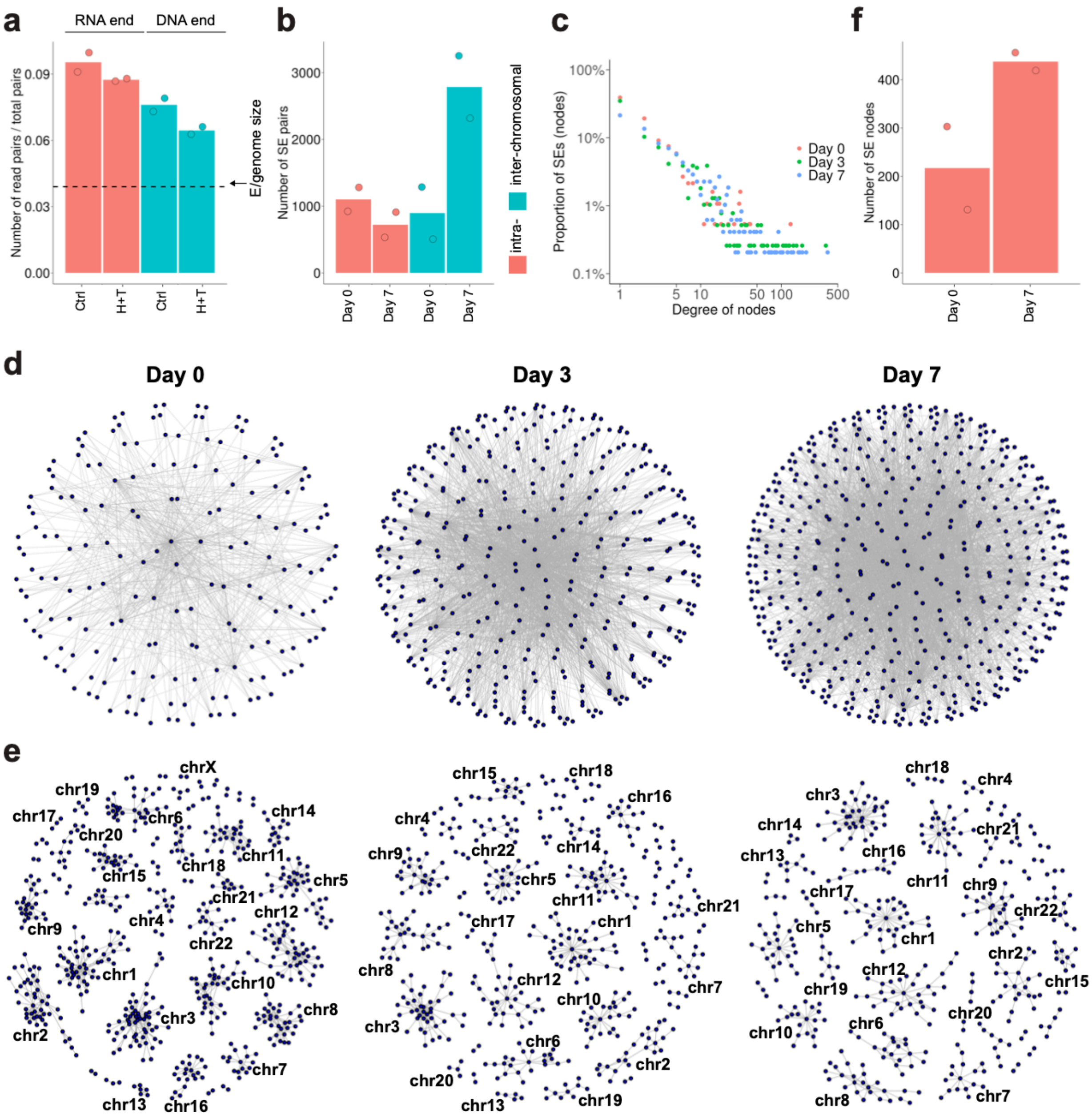

Supplementary Figure 5. **Reciprocal RNA-chromatin interactions between *Linc607SE* and *Serpine1SE*.** (a) Coverage plots of the *Linc607SE* RNA on the genomic region (chr7:80Mb-120Mb) containing the *Serpine1SE* (pink) from Day 0 (red), 3 (green), 7 (blue) ECs. Resolution = 200 kb. Read coverage = (number of read pairs / total pairs) × 10^8. (b) iMARGI contact matrices from RNA on chromosome 7 (rows) to DNA on chromosome 2 (columns). Source data are provided as a Source Data file.

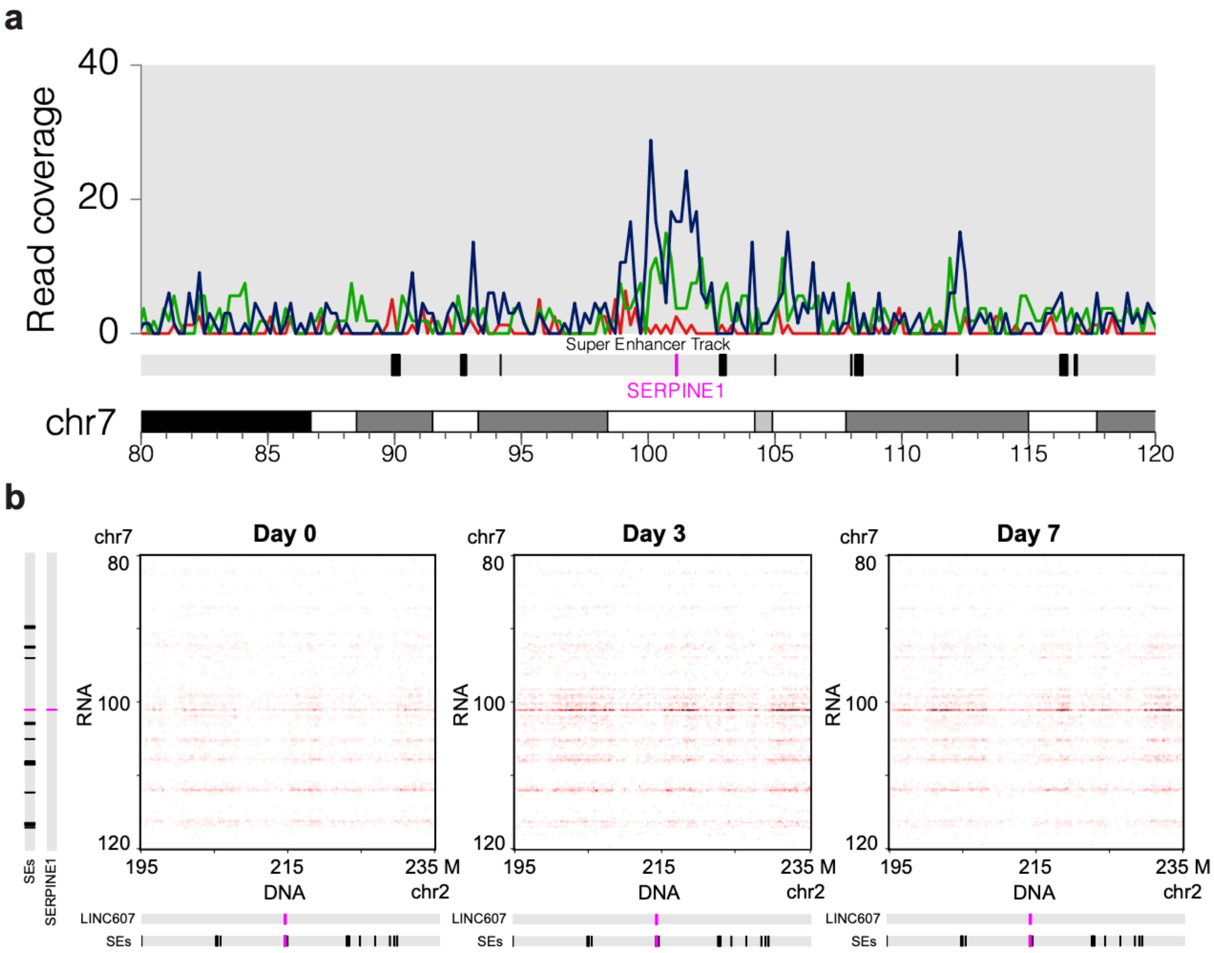

Supplementary Figure 6. Basic information of LINC00607. (a) Genomic feature of *LINC00607*. (b) Exon structure of LINC00607 transcripts (Ensembl). (c) RNA-seq reads of LINC00607 transcripts in HUVECs. The transcript examined in our study is highlighted in red. (d, e) HUVECs were treated as control or with H+T (HT) for 3 days. (d) qPCR of LINC00607 plotted as percentages in subcellular fractions. Data are represented as mean±SEM from 5 independent experiments. (e) RNA FISH images of LINC00607 detected with a LNA probe (QIAGEN) labeled with 5'FAM (green). DRAQ5 was used for nuclear staining (blue). A 5'FAM-labeled probe with scramble sequence was used as a negative control, which did not show any significant signal (in f). Scale bar = 50 µm. The RNA FISH for each condition was performed 3 times independently with similar observation. Representative confocal microscopic images are presented. Source data are provided as a Source Data file.

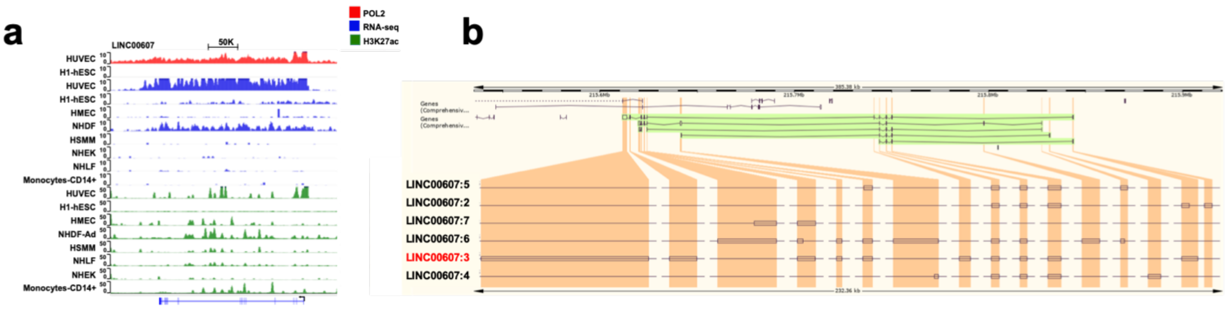

c

Transcript ID	Size (nt)	# of Exons	Relative Expression in HUVECs (ENCODE)							
			Dataset 1: Whole cell (RPKM)		Dataset 1: Cytosol (RPKM)		Dataset1: Nucleus (RPKM)		Dataset 2 (FPKM)	
			rep1	rep2	rep1	rep2	rep1	rep2	rep1	rep2
LINC00607:1	18176	12	NA	NA	NA	NA	NA	NA	NA	NA
LINC00607:2	558	5	0.06	0.05	0.04	0.08	0	0.07	7.5	7
**LINC00607:3**	**3690**	**10**	**5**	**5.04**	**4.41**	**3.85**	**15.51**	**14.24**	**16.78**	**25.86**
LINC00607:4	581	5	0.21	0.81	0.16	0.15	0.15	0.14	11.46	4.74
LINC00607:5	570	5	0.19	0.12	0.11	0.11	0.1	0.09	13.02	22.64
LINC00607:6	2104	9	1.8	2.07	2.45	2.03	4.2	5.52	17.25	19.01
LINC00607:7	526	2	2.63	2.14	1.96	3.81	3.57	3.38	0.47	0.69

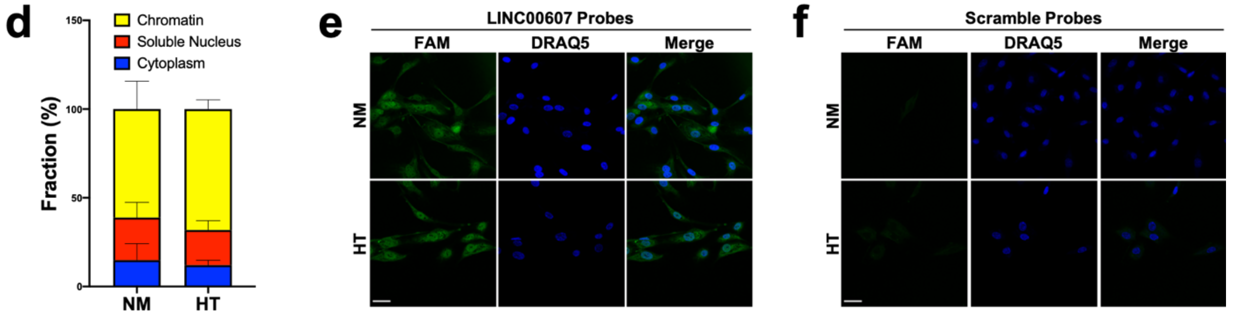

Supplementary Figure 7. Global relaxation of intra-chromosomal DNA contacts in dysfunctional ECs. (a) Distribution of Hi-C derived DNA contact frequencies (y axis) at Day 0 (red), 3 (green), 7 (blue) with respect to genomic distance (x axis). Bin size = 40 kb. (b) Distributions of proportions of reads mapped within TADs. Box plots show the median (center), the interquartile range (IQR, bounds of the box), minima and 75th percentile + 1.5 times IQR (whiskers), and maxima. Bin size = 40 kb. For fair comparisons, 190 million read pairs were randomly sampled from every Hi-C sequencing library for all analyses. Source data are provided as a Source Data file.

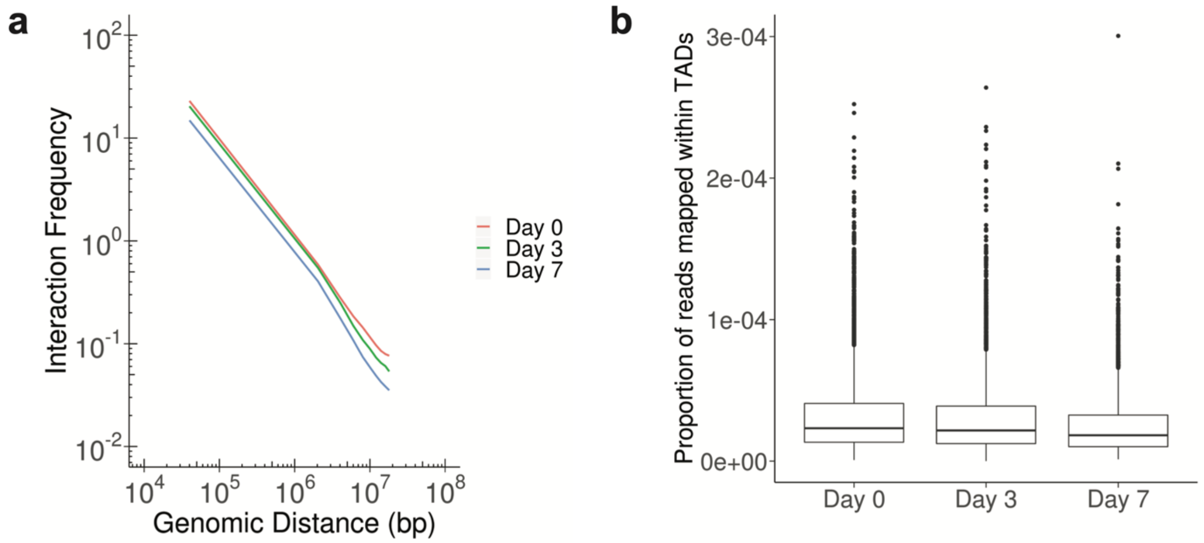

Supplementary Figure 8. *Linc607SE* and *Serpine1SE* DNA FISH. (a) Design of probes targeting chromosomal loci spanning *LINC00607* and *SERPINE1* SEs, as well as the negative control region. (b, c) Distances between *SERPINE1* genomic locus and *LINC00607* or the control region were measured in HUVECs treated with H+T at indicated time points. Representative images (in b) and quantification of DNA FISH based on 290, 285, and 190 cells per each group (in c). Nuclei were stained by DAPI; scale bar = 2 μm. Arrow heads indicate example proximal pair. p-values were generated by non-parametric Wilcoxon-test with Bonferroni correction for multiple comparisons. **** $P < 0.0001$ and * $P < 0.05$. ns denotes not significant. Control = Day 0, D3 = Day 3, D7 = Day 7. Source data are provided as a Source Data file.

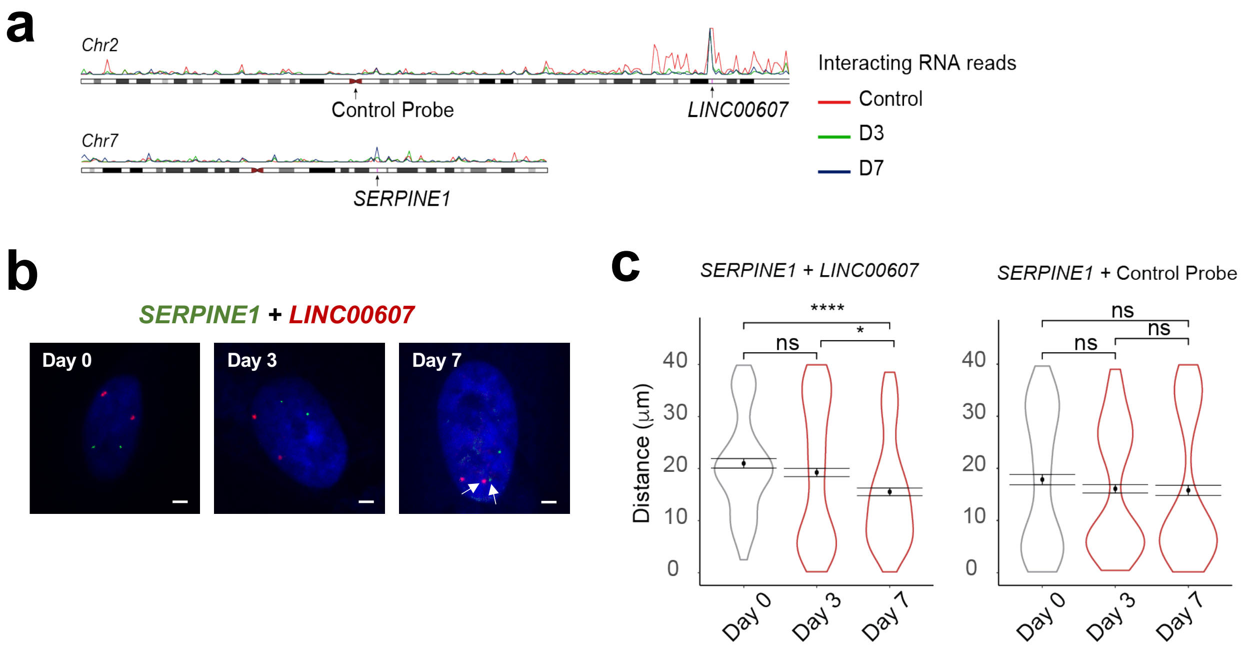

Supplementary Figure 9. Effect of *LINC00607* overexpression (OE) in HUVECs. A LINC00607:3 isoform (most abundant and nucleus-localized transcript in HUVECs) was cloned into a pcDNA3.1(+) vector. HUVECs were transfected with either empty pcDNA3.1(+) plasmid (Empty vector) or plasmid containing *LINC00607* cDNA (0.6 μg per well in 6-well plates). Cells were harvested at 48 h post transfection. RNA levels of respective genes were quantified by qPCR. Data are represented as mean±SEM from 6 independent experiments. **P* =0.0312 based on two-sided Wilcoxon test compared to empty vector control. Source data are provided as a Source Data file.

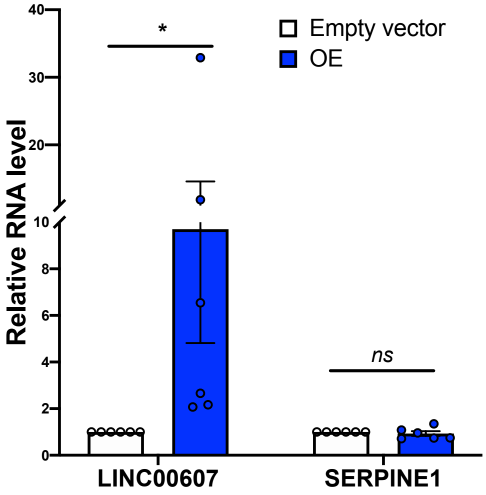

Supplementary Figure 10. Reversal effect of H+T removal in ECs. (a) Morphological changes of HUVECs upon mannitol (N+M 1d) or H+T treatment for 3 days (H+T 3d) followed by incubation in mannitol for 1 day (H3N1) or 3 days (H3N3). (b) Relative RNA levels of indicated mRNAs or LINC00607 under various treatments as shown in (a). Data are represented as mean±SEM from 4 independent experiments. *P = 0.01, 0.04, 0.05, 0.04, 0.03, 0.02, 0.002, 0.03 and 0.04 from left to right in (b) based on two-sided t-test between indicated groups. Source data are provided as a Source Data file.

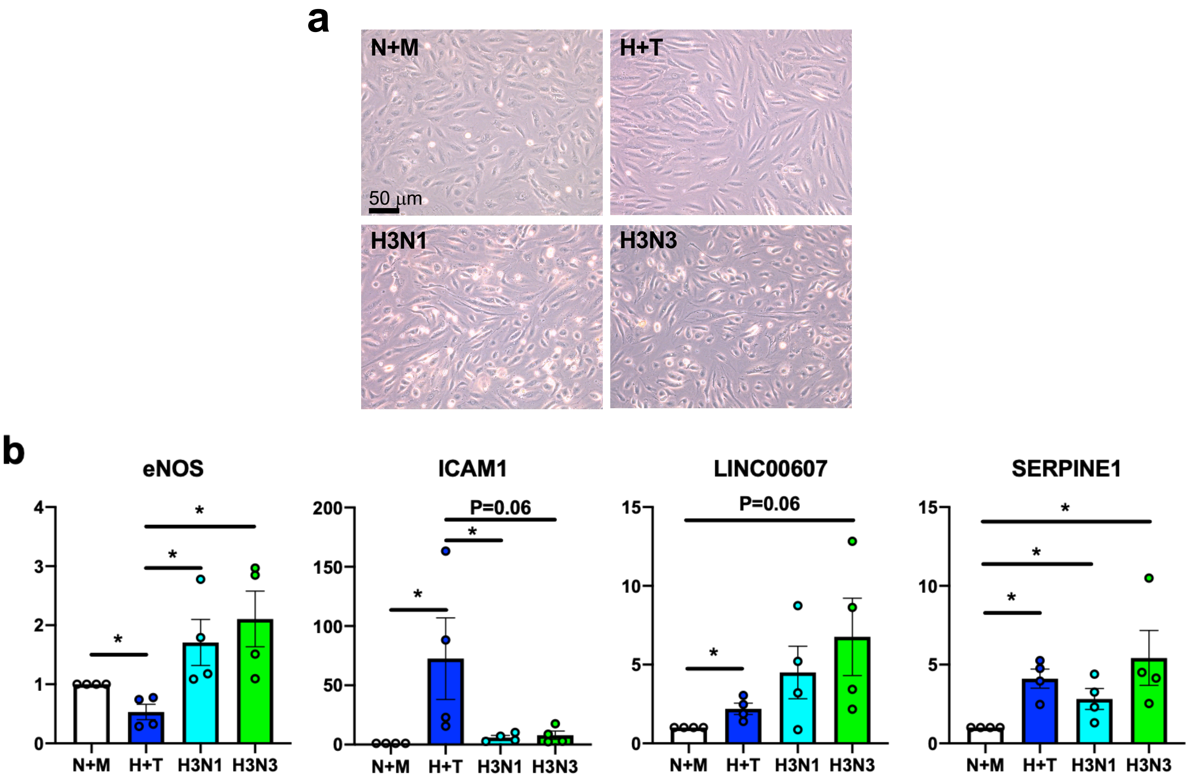

Supplementary Figure 11. scRNA-seq data analysis of human donor-derived ECs. Mesenteric arterial cells were isolated from 2 healthy (n) and 2 type 2 diabetic (T2D) donors for scRNA-seq. Number of cells sequenced are: n.1: 2249, n.2: 1875, T2D.1: 4286, T2D.2, 4405. (a) t-SNE plot showing the entire population of single cells. Cells from each patient type are not completely clustered together given the potential presence of several other cell types besides ECs (for example, smooth muscle cells, macrophages, monocytes, etc.). (b) t-SNE plot showing clusters identified by SNN clustering. (c) EC populations were identified by selecting clusters using *CDH5* expression. Source data are provided as a Source Data file.

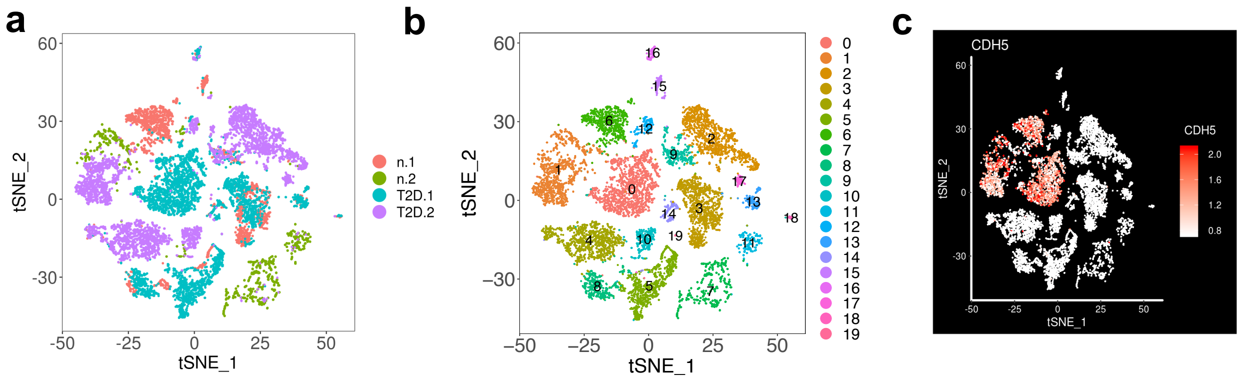

Supplementary Table 1. Enriched pathways in DE genes related to EC dysfunction. Enriched pathway statistical analysis was performed using the functional annotation tool DAVID, which uses a modified one-sided Fisher Exact test. Thus, a modified Fisher Exact P-value is used in order to detect significant enrichment (modified P-value < 0.1).

Term	No. of genes	%	P-value
GO:0007155~cell adhesion	43	5.35	3.32E-06
GO:0001525~angiogenesis	37	4.60	6.02E-12
GO:0006954~inflammatory response	35	4.35	4.24E-05
GO:0006955~immune response	32	3.98	0.00252838
GO:0098609~cell-cell adhesion	31	3.86	2.04E-06
GO:0001666~response to hypoxia	26	3.23	8.91E-08
GO:0030198~extracellular matrix organization	23	2.86	3.73E-05
GO:0016477~cell migration	20	2.49	1.53E-04
GO:0050900~leukocyte migration	17	2.11	6.66E-05
GO:0007568~aging	16	1.99	0.00501572
GO:0007179~transforming growth factor beta receptor signaling pathway	15	1.87	3.60E-05
GO:0033209~tumor necrosis factor-mediated signaling pathway	14	1.74	0.00166058
GO:0006979~response to oxidative stress	13	1.62	0.0026811
GO:0050776~regulation of immune response	13	1.62	0.08217528
GO:0008286~insulin receptor signaling pathway	12	1.49	4.78E-04
GO:0071260~cellular response to mechanical stimulus	11	1.37	8.64E-04
GO:0034097~response to cytokine	10	1.24	3.31E-04
GO:0071356~cellular response to tumor necrosis factor	10	1.24	0.04704454
GO:0007219~Notch signaling pathway	10	1.24	0.05925478
GO:0009749~response to glucose	9	1.12	0.00846146
GO:0050727~regulation of inflammatory response	8	1.00	0.01797915
GO:0050729~positive regulation of inflammatory response	8	1.00	0.03683555
GO:0032869~cellular response to insulin stimulus	8	1.00	0.04706729
GO:0045429~positive regulation of nitric oxide biosynthetic process	7	0.87	0.00965174

GO:0071560~cellular response to transforming growth factor beta stimulus	7	0.87	0.01784758
GO:0007249~I-kappaB kinase/NF-kappaB signaling	7	0.87	0.04320139
GO:0038061~NIK/NF-kappaB signaling	7	0.87	0.06346437
GO:0006006~glucose metabolic process	7	0.87	0.06730356
GO:0070098~chemokine-mediated signaling pathway	7	0.87	0.08397872
GO:0090023~positive regulation of neutrophil chemotaxis	6	0.75	0.00206053
GO:0001974~blood vessel remodeling	5	0.62	0.04675183

Supplementary Table 2. Summary of high-throughput datasets.

Donor ID	Cell type	Assay type	Treatment/Status	# input cells
**N/A**	HUVEC	scRNA-seq	NM control	~ 10,000
		scRNA-seq	NM control	~ 10,000
		scRNA-seq	H+T 3 days	~ 10,000
		scRNA-seq	H+T 3 days	~ 10,000
		scRNA-seq	H+T 7 days	~ 10,000
		scRNA-seq	H+T 7 days	~ 10,000
		Hi-C	NM control	~ 1 million
		Hi-C	H+T 3 days	~ 1 million
		Hi-C	H+T 7 days	~ 1 million
		iMARGI	NM control	~ 3 million
		iMARGI	NM control	~ 3 million
		iMARGI	H+T 3 days	~ 3 million
		iMARGI	H+T 7 days	~ 3 million
		iMARGI	H+T 7 days	~ 3 million
**HC-1**	Mesenteric arterial EC	scRNA-seq	Healthy	~ 10,000
**HC-1**		scRNA-seq	Healthy	~ 10,000
**T2D-1**		scRNA-seq	Dysfunctional	~ 10,000
**T2D-2**		scRNA-seq	Dysfunctional	~ 10,000

Supplementary Table 3. H+T-induced SE hubs. The ordered number of the SE (Index), the first time that this SE becomes a hub in the H+T treatment time course (Emerging time point), and the genes contained in the SE, including coding, lincRNA, and pseudogene genes. The genes known to be related to EC dysfunction are marked in red.

Index	Emerging time point	Genes contained in this SE
**557**	Day 0	MALAT1; AP000769.7; AP000769.1
**23**	Day 3	MACF1
**216**	Day 3	FNDC3B
**253**	Day 3	TRIO; AC016549.1
**391**	Day 3	TRIM56; SERPINE1
**438**	Day 3	EXT1
**442**	Day 3	PVT1
**469**	Day 3	PALM2; PALM2-AKAP2; AKAP2
**503**	Day 3	N/A
**548**	Day 3	RNA5SP335; NAV2-AS3
**556**	Day 3	NEAT1
**682**	Day 3	THBS1
**692**	Day 3	SMAD3
**840**	Day 3	RUNX1; AF015262.2; AF015720.3; AP000687.1
**217**	Day 7	NLGN1
**219**	Day 7	EIF2B5; DVL3; AP2M1; ABCF3; VWA5B2; ALG3; ECE2; CAMK2N2; PSMD2; EIF4G1; FAM131A; CLCN2; POLR2H; THPO; CHRD; RP11-433C9.2; EPHB3
**264**	Day 7	PDE4D; NDUFB4P2; SETP21; PART1
**328**	Day 7	CASC15; NBAT1
**470**	Day 7	PALM2; PALM2-AKAP2; AKAP2
**583**	Day 7	VWF
**632**	Day 7	NCOR2; RP11-83B20.1
**658**	Day 7	SAMD4A
**694**	Day 7	THSD4; CT62
**733**	Day 7	ANKRD11
**794**	Day 7	SIPA1L3

Supplementary Table 4. Information of donors for human mesenteric arterial EC isolation.

Donor ID	HbA1c	Donor info
HC-1	5.4%	Caucasian, Male, 59, BMI 28.2
HC-2	5.5%	Hispanic, Male, 28, BMI 21.0
T2D-1	9.5%	Hispanic, Female, 42, BMI 40.3;<br>T2D> 10yr
T2D-2	6.9%	Hispanic, Male, 46, BMI 45.8;<br>Untreated T2D

Supplementary Table 5. List of genes embedded in emergent LINC00607-interacting SEs and of which expression is suppressed in H+T-treated ECs with LINC00607 LNA1. Y/N in the last column indicates the embedded hub is or is not a hub SE.

SE Index	Gene	Hub SE
366	PRKAR1B	N
461	TRMT10B	N
506	CUBN	N
253	TRIO	Y
391	TRIM56	Y
391	SERPINE1	Y
652	COL4A1	N
652	COL4A2	N
694	THSD4	Y
794	SIPA1L3	Y
279	ERAP1	N
328	CASC15	Y
222	LPP	N
632	NCOR2	Y

Supplementary Table 6. Sequences of qPCR primers, LNA GapmeRs, information of DNA and RNA FISH probes.

qPCR primers	Forward	Reverse
**LINC00607**	ACCGGGCGTTGAGAATACAA	ACACTTGGCGAAACTTCCCT
**eNOS**	TGATGGCGAAGCGAGTGAAG	ACTCATCCATACACAGGACCC
**ICAM1**	GTGTCCTGTATGGCCCCCGACT	ACCTTGCGGGTGACCTCCCC
**ACTB**	CATGTACGTTGCTATCCAGGC	CTCCTTAATGTCACGCACGAT
**SERPINE1**	AGTGGACTTTTCAGAGGTGGA	GCCGTTGAAGTAGAGGGCATT
**α-SMA**	CAGGGCTGTTTTCCCATCCAT	GCCATGTTCTATCGGGTACTTC

LNAs	Sequence	Position (NR_037195.1)
**LNA-1**	ATAGGTCACGCATTCT	210-225
**LNA-2**	CAACTGTGGAATGATA	2033-2048
**Scramble**	AACACGTCTATACGC	

SKU	DNA FISH Probes	Chromosome coordinate (Hg38)
**CLN-1008**	Clone Library: RPCI-11<br>Clone Name: 946O2<br>Dye Color: Orange 5-TAMRA dUTP	Chr2:216224988-216402549
**CLN-1004**	Clone Library: RPCI-11<br>Clone Name: 954P20<br>Dye Color: Green 5-Fluorescein dUTP	Chr7:100405127-100578561
**CHR02-10-RE**	Chromosome 02 Control Probe<br>Dye Color: Red 5-ROX dUTP	Chr2:93900000-96000000

RNA FISH Probe	Sequence	Position (NR_037195.1)
**LINC00607 probe**	ATAGGTCACGCATTCT	210-225
