# Supplementary material for: Stress-induced RNA–chromatin interactions promote endothelial dysfunction
Source: Nat Commun. 2020 Oct 15;11:5211. doi: 10.1038/s41467-020-18957-w (PMC7566596; doi:10.1038/s41467-020-18957-w)
Supplement: Supplementary file 3 — Reporting Summary [file 41467_2020_18957_MOESM3_ESM.pdf]

nature research

Corresponding author(s): Zhen Bouman Chen and Sheng Zhong

Last updated by author(s): Aug 24, 2020

Reporting Summary

Nature Research wishes to improve the reproducibility of the work that we publish. This form provides structure for consistency and transparency in reporting. For further information on Nature Research policies, see our Editorial Policies and the Editorial Policy Checklist.

Statistics

For all statistical analyses, confirm that the following items are present in the figure legend, table legend, main text, or Methods section.

n/a	Confirmed	
☐	☒	The exact sample size (*n*) for each experimental group/condition, given as a discrete number and unit of measurement
☐	☒	A statement on whether measurements were taken from distinct samples or whether the same sample was measured repeatedly
☐	☒	The statistical test(s) used AND whether they are one- or two-sided<br>*Only common tests should be described solely by name; describe more complex techniques in the Methods section.*
☒	☐	A description of all covariates tested
☐	☒	A description of any assumptions or corrections, such as tests of normality and adjustment for multiple comparisons
☐	☒	A full description of the statistical parameters including central tendency (e.g. means) or other basic estimates (e.g. regression coefficient) AND variation (e.g. standard deviation) or associated estimates of uncertainty (e.g. confidence intervals)
☐	☒	For null hypothesis testing, the test statistic (e.g. *F*, *t*, *r*) with confidence intervals, effect sizes, degrees of freedom and *P* value noted<br>*Give P values as exact values whenever suitable.*
☒	☐	For Bayesian analysis, information on the choice of priors and Markov chain Monte Carlo settings
☒	☐	For hierarchical and complex designs, identification of the appropriate level for tests and full reporting of outcomes
☒	☐	Estimates of effect sizes (e.g. Cohen's *d*, Pearson's *r*), indicating how they were calculated

Our web collection on statistics for biologists contains articles on many of the points above.

Software and code

Policy information about availability of computer code

Data collection

iMARGI: Illumina HiSeq 4000 was used for sequencing, and Illumina Casava (v1.8) for base calling. Paired-end read pairs duplicates were removed using FastUniq (v1.1). Paired-end read pairs were aligned to the hg38 genome assembly using STAR (v2.5.4b). Samtools (v1.6) and in-house scripts (https://github.com/Zhong-Lab-UCSD/NCOMMS-19-24818) were used to parse the mapped reads.

Hi-C: Hi-C was performed using an Arima-HiC kit (Arima Genomics, Inc.) following the manufacturer's manual. Illumina HiSeq 4000 was used for sequencing and Illumina Casava (v1.8) for base calling. Data were collected and processed using the publicly available software HiCtool (v2.2) (https://github.com/Zhong-Lab-UCSD/HiCtool
). Paired-end read pairs were aligned using Bowtie 2 (v2.2.9). Samtools (v1.6), HiFive (v1.4) and in-house scripts (all available in HiCtool) were used to pre-truncate, parse, filter, deduplicate the mapped read pairs.

scRNA-seq: 10X Genomics Single Cell 3' v3 gene expression was used to generate the single-cell RNA-seq library. Illumina HiSeq 2500 (in-vitro data) and HiSeq 4000 (in-vivo data) were used for sequencing. 10X Genomics cellranger (v3.0) was used to align the sequencing data, using the hg38 reference transcriptome file refdata-cellranger-GRCh38-3.0.0 provided by 10X Genomics.

RNA-seq: KAPA mRNA HyperPrep Kit (Roche Diagnostics) was used to generate the stranded polyA-enriched RNA-seq library following the manufacturer's manual. Illumina HiSeq 2500 was used for sequencing with the SR50 mode. Data were aligned to the hg38 reference genome using STAR (v2.5.4b).

Data analysis

iMARGI: Data were analyzed using in-house R scripts (https://github.com/Zhong-Lab-UCSD/NCOMMS-19-24818), exploiting several packages, such as: GenomicRanges (v1.36.0), GenomicAlignments (v1.20.2), gdata (v2.18.0) as the main packages for genomic data manipulation; ggbio (v1.32.0), KaryoploteR (v1.10.5), Gviz (v1.28.3) for visualization of genomic data; igraph (v1.2.4.1) and Cytoscape (v3.5.1) for network analysis and visualization. A Python (2.7) script was used for plotting the heatmaps, extracted and re-adapted from HiCtool (v2.2) to handle non-symmetrical contact matrices.

Hi-C: Data were analyzed using the software HiCtool (v2.2) (https://github.com/Zhong-Lab-UCSD/HiCtool). HiCtool exploits HiFive (v1.4) and Hi-Corrector (v1.2) to generate the contact matrices and normalize the data. Matplotlib (v1.5.1) was used for plotting the heatmaps.

scRNA-seq: Data were analyzed and visualized using in-house scripts (https://github.com/Zhong-Lab-UCSD/NCOMMS-19-24818) based on functions and algorithms (PCA, t-SNE, Wilcoxon test, etc.) from the R package Seurat (v2.3.4). Pathway enrichment analysis for differentially expressed genes was performed using the Database for Annotation, Visualization and Integrated Discovery (DAVID, https://david.ncifcrf.gov/).

RNA-seq: featureCounts from the Subread package (v2.0.0) was used to count the number of features (uniquely mapped reads) over the genes. The output raw count matrices (genes-by-samples) were used as input data for the analysis performed with the R package DESeq2 (v1.24.0).

For manuscripts utilizing custom algorithms or software that are central to the research but not yet described in published literature, software must be made available to editors and reviewers. We strongly encourage code deposition in a community repository (e.g. GitHub). See the Nature Research guidelines for submitting code & software for further information.

Data

Policy information about availability of data

All manuscripts must include a data availability statement. This statement should provide the following information, where applicable:

- Accession codes, unique identifiers, or web links for publicly available datasets
- A list of figures that have associated raw data
- A description of any restrictions on data availability

All high-throughput data supporting the current study are accessible on GEO (accession number GSE135357). Ensembl annotation data GRCh38.84 (Homo_sapiens.GRCh38.84.gtf.gz) are publicly available at ftp://ftp.ensembl.org/pub/release-84/gtf/homo_sapiens/Homo_sapiens.GRCh38.84.gtf.gz. HUVEC super enhancer data (HUVEC.bed) were downloaded from dbSUPER (https://asntech.org/dbsuper/data/bed/hg19/HUVEC.bed). HUVEC enhancer data (HUVEC.fasta) were downloaded from EnhancerAtlas (http://enhanceratlas.org/data/enhseq/HUVEC.fasta). Other data are available from the corresponding authors upon reasonable request. Source data are provided in this paper.

Field-specific reporting

Please select the one below that is the best fit for your research. If you are not sure, read the appropriate sections before making your selection.

☒ Life sciences ☐ Behavioural & social sciences ☐ Ecological, evolutionary & environmental sciences

For a reference copy of the document with all sections, see nature.com/documents/nr-reporting-summary-flat.pdf

Life sciences study design

All studies must disclose on these points even when the disclosure is negative.

Sample size

Sample size was determined based on our previous studies using HUVECs and various methods used in this manuscript. References include Chen et al. Circulation 2015, Miao et al Nat Commun 2018, Sridhar et al Curr Biol 2017.

Data exclusions

No data were excluded.

Replication

For iMARGI, 3 sets of HUVECs were prepared and 2 sets of libraries were prepared and passed in-house QC. RNA-seq and scRNA-seq were performed with two biological replicates and all data were reported. For experiments presented in Figures 1 and 4, 4-8 independent experiments were performed by two independent researchers as stated in the figure legends.

Randomization

The culture HUVECs were plated in multiple culture dishes. Cells at comparable confluence were randomly selected for various treatment, including mannitol vs high-glucose + TNFa, or scramble vs LNAs.
For donor-derived monocytes, 4 healthy donors were randomly selected.
For donor-derived ECs, 2 healthy and 2 T2D donors that fit into our inclusion criteria based on HbA1c level and known medical record were randomly selected.

Blinding

(Immuno)fluorescent staining, DNA FISH and RNA FISH were performed in a single blinded fashion. Other experiments were not done in a blind fashion as the investigators need to design, conduct, and analyze the data, thus they need to know the identification of samples.

Reporting for specific materials, systems and methods

We require information from authors about some types of materials, experimental systems and methods used in many studies. Here, indicate whether each material, system or method listed is relevant to your study. If you are not sure if a list item applies to your research, read the appropriate section before selecting a response.

Materials & experimental systems

n/a	Involved in the study
☐	☒ Antibodies
☐	☒ Eukaryotic cell lines
☒	☐ Palaeontology and archaeology
☒	☐ Animals and other organisms
☐	☒ Human research participants
☒	☐ Clinical data
☒	☐ Dual use research of concern

Methods

n/a	Involved in the study
☒	☐ ChIP-seq
☒	☐ Flow cytometry
☒	☐ MRI-based neuroimaging

Antibodies

Antibodies used

Mouse anti-human α-SMA antibody (Abcam, ab124964) and rabbit anti-VE-cadherin antibody (Abcam, ab33168); Alexa Fluor 488-conjugated goat anti-mouse (for α-SMA staining, Fisher, A10680) and Alexa Fluor 555-conjugated donkey anti-rabbit (for VE-cadherin staining, Fisher, A31572) antibody. For monocyte isolation, CD14 microBeads conjugated to monoclonal anti- human CD14 antibodies (Miltenyi Biotec, 130-050-201) was used.

Validation

All the antibodies used in this study have been validated by the vendors as indicated on the websites. Citations are listed as below:

Mouse anti-human α-SMA antibody (Abcam, ab124964) validate in human for immunofluorescence staining:
1. Kim JE et al. Single cell and genetic analyses reveal conserved populations and signaling mechanisms of gastrointestinal stromal niches. Nat Commun 11:334 (2020).
2. Dong S et al. Monitoring spatiotemporal changes in chaperone-mediated autophagy in vivo. Nat Commun 11:645 (2020).

Rabbit anti-VE-cadherin antibody (Abcam, ab33168) validated in human for immunofluorescence staining:
1. Dong W et al. Mesenchymal-endothelial transition-derived cells as a potential new regulatory target for cardiac hypertrophy. Sci Rep 10:6652 (2020).
2. Martinez-Sanchez J et al. Acute Graft-vs.-Host Disease-Associated Endothelial Activation in vitro Is Prevented by Defibrotide. Front Immunol 10:2339 (2019).

Alexa Fluor 488-conjugated goat anti-mouse antibody (for α-SMA staining, Fisher, A10680) validated in mouse and human for Immunocytochemistry:
1. Lalit, Pratik A et al. Generation of multipotent induced cardiac progenitor cells from mouse fibroblasts and potency testing in ex vivo mouse embryos. Nature protocols vol. 12,5: 1029-1054 (2017).
2. Usenovic M, et al. Internalized Tau Oligomers Cause Neurodegeneration by Inducing Accumulation of Pathogenic Tau in Human Neurons Derived from Induced Pluripotent Stem Cells. J Neurosci. 2015;35(42):14234-14250.

Alexa Fluor 555-conjugated donkey anti-rabbit antibody (for VE-cadherin staining, Fisher, A31572) validated in mouse for immunolabeling:
1. Lafragette, Audrey et al. Reduction of Cocaine-Induced Locomotor Effects by Enriched Environment Is Associated with Cell-Specific Accumulation of ΔFosB in Striatal and Cortical Subregions. The international journal of neuropsychopharmacology vol. 20,3: 237-246 (2017).

CD14 microBeads validated in human for monocyte isolation:
1. Verreck, F. A. et al. (2004) Human IL-23-producing type 1 macrophages promote but IL-10-producing type 2 macrophages subvert immunity to (myco)bacteria. Proc. Natl. Acad. Sci. U.S.A. 101: 4560-4565
2. Vitale, S. et al. (2004) Soluble fractalkine prevents monocyte chemoattractant protein-1-induced monocyte migration via inhibition of stress-activated protein kinase 2/p38 and matrix metalloproteinase activities. J. Immunol. 172: 585-592

Eukaryotic cell lines

Policy information about cell lines

Cell line source(s)

Human Umbilical Vein Endothelial Cells (HUVECs) were purchased from Cell Applications, Inc. with Catalog #: 200p-05n. Human monocytes were isolated from healthy donors

Authentication

Human monocytes were authenticated using PCR-based gene expression profiling and FACS for monocyte markers as well as cytokine-induced phagocytosis assays. HUVECs have been pre-screened to demonstrate stimulation-dependent angiogenesis and key EC signaling pathways. We have also routinely tested for the human EC marker genes using PCR and immunofluorescent staining.

Mycoplasma contamination

The cells have been tested negative for mycoplasma contamination.

Commonly misidentified lines
(See ICLAC register)

No commonly misidentified cell lines were used in the study.

Human research participants

Policy information about studies involving human research participants

Population characteristics	For monocyte isolation, donors were randomly selected from healthy donors based on existing medical record and self report regardless age, gender, ethnicity. For EC isolation, donors were all deceased organ donors. We randomly selected donors of 40-60 years old, regardless gender and ethnicity, that fit into healthy or T2D categories based on predominantly HbA1c level and medical records.
Recruitment	The study does not involve any active recruitment of participants.
Ethics oversight	Institutional Review Board of City of Hope

Note that full information on the approval of the study protocol must also be provided in the manuscript.
